# Responses of metastatic basal cell and cutaneous squamous cell carcinomas to anti-PD1 monoclonal antibody REGN2810

**DOI:** 10.1186/s40425-016-0176-3

**Published:** 2016-11-15

**Authors:** Gerald S. Falchook, Rom Leidner, Elizabeth Stankevich, Brian Piening, Carlo Bifulco, Israel Lowy, Matthew G. Fury

**Affiliations:** 1 Sarah Cannon Research Institute at HealthONE, Denver, CO USA; 2 Department of Medicine, Providence Portland Medical Center, Portland, OR USA; 3 Oncology Clinical Sciences, Regeneron Pharmaceuticals, 777 Old Saw Mill River Road, Tarrytown, NY 10591-6707 USA; 4 Department of Pathology, Providence Portland Medical Center, Portland, OR USA

**Keywords:** Basal cell carcinoma, Cutaneous squamous cell carcinoma, Mutation burden, REGN2810, Phase 1, Programmed Death-1, Immune checkpoint inhibitor

## Abstract

**Background:**

Basal cell carcinoma (BCC) and cutaneous squamous cell carcinoma (CSCC) share exposure to UV light as the dominant risk factor, and these tumors therefore harbor high mutation burdens. In other malignancies, high mutation burden has been associated with clinical benefit from therapy with antibodies directed against the Programmed Death 1 (PD-1) immune checkpoint receptor. Highly mutated tumors are more likely to express immunogenic tumor neoantigens that attract effector T cells, which can be unleashed by blockade of the PD-1 immune checkpoint.

**Case presentations:**

This report describes a patient with metastatic BCC and a patient with metastatic CSCC who were treated with REGN2810, a fully human anti-PD-1 monoclonal antibody, in an ongoing phase 1 trial (NCT02383212). The CSCC patient has experienced an ongoing complete response (16+ months), and the BCC patient has experienced an ongoing partial response (12+ months).

**Conclusions:**

These case reports suggest that UV-associated skin cancers, beyond melanoma, are sensitive to PD-1 blockade.

**Trial registration:**

Clinicaltrials.gov NCT02383212. Registered 2 February 2015.

## Background

Basal cell carcinoma (BCC) and cutaneous squamous cell carcinoma (CSCC) share exposure to UV light as the dominant risk factor, and these tumors therefore harbor high mutation burdens [1–3]. In other malignancies, high mutation burden has been associated with clinical benefit from therapy with antibodies directed against the Programmed Death 1 (PD-1) immune checkpoint receptor [4]. Highly mutated tumors are more likely to express immunogenic tumor neoantigens that attract effector T cells, which can be unleashed by blockade of the PD-1 immune checkpoint [5]. This report describes a patient with metastatic BCC and a patient with metastatic CSCC who were treated with REGN2810, a fully human anti-PD-1 monoclonal antibody in an ongoing phase 1 trial (NCT02383212).

## Case presentation 1

### Basal cell carcinoma

The patient is a 66 year-old woman who was diagnosed with a primary desmoplastic BCC arising on the left aspect of the chin in April 2008, which was resected with Mohs surgery. A localized recurrence in the same location was identified in June 2010, and a wide local excision revealed invasion into the left mandible and involvement of one out of 18 lymph nodes. The patient received adjuvant radiation and remained in remission until 2014, when enlarging lung nodules observed on surveillance chest imaging were biopsied and confirmed the presence of metastatic BCC. The patient subsequently received the Hedgehog pathway inhibitor (HHI) vismodegib from September 2014 until February 2015. She initially responded but discontinued because of progressive disease.

**Fig. 1:**
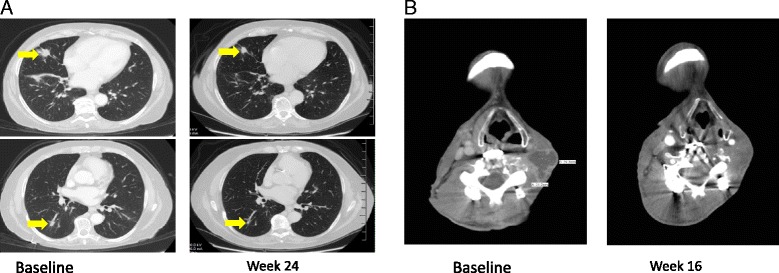
**a** Lung metastases in BCC patient indicated by yellow arrows at baseline (upper, 1.6 cm; lower 1.4 cm), *left*, and at Week 24 (upper, 1.3 cm; lower, 0.6 cm), *right*. **b** Neck mass in CSCC patient at baseline (3.1 cm), *left*, and at Week 16 (1.6 cm), *right*. The lesions shown are the only target lesions for both patients

In August 2015 after continued slow progression, the patient enrolled on the phase 1 study of REGN2810 to a cohort receiving 10 mg/kg IV every 2 weeks, and received her first dose on August 3, 2015. Two lung metastases were followed as target lesions. The patient has not experienced any treatment-related adverse events of grade 1 or higher. Response assessments at the end of 8 weeks (3 % increase) and 16 weeks (10 % decrease) demonstrated stable disease by RECIST criteria. The response assessment at the end of 24 weeks demonstrated a reduction in target lesion measurements of 37 % (Fig. 1a), and this was confirmed at 32 weeks. Two sub-centimeter non-target lesions in the lung were stable (not shown). The patient completed the planned 48 weeks of protocol treatment with REGN2810 on June 20, 2016, and began post-treatment follow up with her medical oncologist. As of most recent radiology assessment on August 3, 2016, partial response has been maintained and the patient continues in post-treatment follow-up (12+ months).

Next generation DNA sequencing (NGS) of an archived recurrent disease tumor sample was performed at Foundation Medicine, Inc (315 gene panel, FoundationOne™). This identified 14 genetic alterations (*PTCH1* Y334*, *ERBB4* E317K, *KIT* M882I, *ATR* R668W, *GRM3* S154F, *MYCN* P44F, *TP53* P177L, *TP53* Q100*, *TP53* S215N, *FGF6* E119K, *GRIN2A* R1067W,*INHBA* R306C, *PPP2R1A* P179L, and *TERT* promoter -124C > T) that were predicted not to be present in germline. Immunohistochemistry (IHC) analysis of PD-L1 expression in this sample was negative (no staining) in tumor and infiltrating immune cells, using the rabbit anti-human clone SP142 (Spring Bioscience, Pleasanton, CA) and the BenchMark XT automated system (Ventana Medical Systems, Inc., Tucson, AZ).

## Case presentation 2

### Cutaneous squamous cell carcinoma

The patient is a 52 year-old man who was diagnosed with cutaneous squamous cell carcinoma of the left cheek in May 2002. He underwent Mohs surgery with clear margins. He experienced multiple recurrences, and underwent at least 9 additional Mohs surgeries. He underwent wide local excision over left mandible in April 2006, and left parotidectomy in February 2008. Adjuvant RT was administered to left cheek (February - April 2005), left mandible (May 2006), left neck (with concurrent cetuximab, April - June 2008), and bilateral neck (with concurrent carboplatin, April - May 2010). Other systemic therapies were capecitabine (March - April 2008), and cisplatin + docetaxel (February - March 2010). On November 14, 2012 he underwent excision with clear margins for a 2.2 cm in-scar recurrence of the left neck. On February 18, 2015, invasive CSCC at C4-C5 vertebral bodies necessitated emergent decompression of cervical spinal cord with C4-C5 anterior corpectomy and C4-C6 posterior laminectomy.

In March 2015, he was enrolled on the phase 1 study in the first cohort, receiving 1 mg/kg REGN2810 every 2 weeks by vein. First dose was March 30, 2015. The patient generally tolerated treatment well, with transient grade 1 skin rash, one day of grade two shaking chills and grade 1 flu-like symptoms, and grade 2 lymphopenia all deemed related to study drug. Response at Week 16 is shown in Fig. 1b. Complete radiologic response of the left neck lesion was achieved at Week 40. The patient completed the planned 48 weeks of protocol treatment with REGN2810 on February 15, 2016. He continues on post-treatment follow up with his medical oncologist without clinical or radiographic evidence of disease recurrence at the most recent radiology assessment (16+ months) on August 8, 2016.

A formalin-fixed paraffin-embedded (FFPE) block was prepared from the C4-C5 CSCC corpectomy specimen and NGS (50 gene panel, Ion AmpliSeq V2) was performed at the treating physician’s institution, along with matched germline sequencing from blood. NGS revealed a spectrum of somatic mutations (*TP53* Q100X, *FBXW7* G437E, *CTNNB1* H36Y, *PIK3CA* L113F, *KDR* S1200F) that were present in tumor but not matched normal blood.

## Discussion

We report the first confirmed partial response in a patient with metastatic BCC treated with a PD-1 inhibitor (REGN2810), as well as an ongoing complete response in a patient with metastatic CSCC. The deep and sustained responses of these heavily pretreated patients to anti-PD-1 monotherapy in this phase 1 study are consistent with the hypothesis that high mutation burdens in BCC and CSCC elicit antitumor cellular immunity that could be unleashed by blockade of the PD-1/PD-L1 checkpoint pathway. Independent lines of evidence support the study of PD-1 blockade in BCC and CSCC. The tumor microenvironment of UV-induced tumors is immunosuppressive, as initially described in murine models of UV-induced tumors in the 1980s [6]. The adaptive cellular immune system plays a critical role in surveillance and eradication of CSCC and BCC, as evidenced by the increased risks of these cancers in solid organ transplant recipients on immunosuppressive therapy: greater than 65-fold for CSCC and 10-fold for BCC [7]. Activation of the innate immune system can also eradicate UV-associated tumors as seen with imiquimod, a Toll-like receptor - 7 agonist that is highly active against small superficial BCCs [8]. Imiquimod is associated with induction of peritumoral infiltration by CD8^+^ T cells [9].

There are several recent case reports of dramatic responses in patients with advanced CSCCs treated with the PD-1 inhibitors pembrolizumab or nivolumab [10–13]. A recent report also described a patient with metastatic BCC treated for 4 cycles with pembrolizumab off label [13], who experienced initial disease progression followed by stabilization of lung metastases over an 11 month period, accompanied by development of several new cutaneous BCC lesions that were excised during this interval. This patient did not experience an objective partial response by RECIST, as observed in the BCC patient in our report.

NGS results were obtained from targeted exome panels performed on archived tumor samples from both the BCC patient (14 alterations among 315 genes) and the CSCC patient (5 alterations among 50 genes) in Case 1 and Case 2, respectively. In consideration of the recent observation that it may be possible to extrapolate from targeted exome panels to estimate total mutation counts in whole exomes [1, 14], it is likely that both cases harbor mutation burdens that are at least moderately high. Ongoing and planned clinical studies with REGN2810 will allow exploration of potential associations between mutation burden, PD-L1 expression, and efficacy in BCC and CSCC.

The clinical observations in the BCC patient and the CSCC patient in this report converge with recent clinical observations regarding the efficacy of anti-PD-1 or anti-PD-L1 therapy in Merkel cell carcinoma (MCC), a less common non-melanoma skin cancer. Pembrolizumab achieved a response rate of 56 % (14/25) in patients with unresectable MCC who had not received prior systemic therapy [15]. Avelumab, an anti-PD-L1 IgG1 monoclonal antibody, yielded objective responses in 32 % (28/88) in patients with metastatic MCC that had been treated with at least one prior regimen [16]. Although the mechanism of immune response in MCC requires further study, T cells may recognize viral antigen due to the presence of Merkel cell polyoma virus in the majority of these tumors, or may recognize neo-antigens arising in the context of high mutation burden due to UV damage in MCC tumors that are not virus-associated [17].

Among the first 60 patients treated in this phase 1 study of REGN2810, one partial response was observed among 2 MCC patients and this response is ongoing at 13+ months. The response was seen in non-irradiated target lesions in an MCC patient who was treated with 3 mg/kg REGN2810 every two weeks, plus a single course of hypofractionated radiation therapy (6 Gy/fraction X 5 fractions) to a non-target lesion [18]. Also among the first 60 patients, a metastatic BCC patient enrolled to receive 3 mg/kg REGN2810 every two weeks plus a single course of hypofractionated radiotherapy (6 Gy X 5 fractions to a non-target lesion) plus low dose cyclophosphamide, and this patient has stable disease at the most recent response assessment (16+ weeks on study). In summary, there have been 3 objective responses among the 5 non-melanoma skin cancer patients enrolled in the dose escalation portion of the phase 1 study [18].

Mechanisms of anti-tumor immune response may differ among non-melanoma skin cancers. For example, baseline intratumoral infiltration with CD8+ T cells may be lower in untreated BCC than in CSCC [9]. In BCC, treatment with hedgehog inhibitors has been associated with an influx of cytotoxic T cells and activation of adaptive immune functions [19]. A question for further study is whether prior treatment with a hedgehog inhibitor, as occurred in the BCC patient in this case report, can potentiate anti-tumor immune responses in the context of subsequent anti-PD-1 therapy and if the interval since prior hedgehog inhibitor therapy is important.

## Conclusions

Here we describe a radiographic complete response in a CSCC patient and the first reported confirmed partial response in a BCC patient treated with the anti-PD-1 monoclonal antibody REGN2810. A general principle appears to be that UV-associated skin cancers beyond melanoma are sensitive to PD-1 blockade. A reductionist model would predict that UV-associated tumors with higher load of non-synonymous mutations will be more responsive to PD-1 blockade than those with lower mutation load. However, numerous other variables must be considered, including tumor clonality, the distributions and expression patterns of effector T cells and other immune cells in the tumor microenvironment, and T cell receptor repertoire. The accessibility of UV-associated skin tumors for biopsy should allow clinical studies of patients with these cancers to illuminate these core concepts in immuno-oncology. An ongoing phase 2 trial will estimate the overall response rate of REGN2810 in patients with unresectable or metastatic CSCC (NCT02760498), and a similar study of REGN2810 in BCC is planned for patients who have been previously treated with hedgehog pathway inhibitors.

## Abbreviations

C4-C6: 4th through 6th cervical vertebrae
Gy: Gray
kg: Kilogram
mg: Milligram
NGS: Next generation DNA sequencing
PD-L1: Programmed death-ligand 1
UV: Ultraviolet light
